# Multi-frequency tapping-mode atomic force microscopy beyond three eigenmodes in ambient air

**DOI:** 10.3762/bjnano.5.175

**Published:** 2014-09-25

**Authors:** Santiago D Solares, Sangmin An, Christian J Long

**Affiliations:** 1 Department of Mechanical Engineering, University of Maryland, College Park, Maryland 20742, United States; current address: Department of Mechanical and Aerospace Engineering, George Washington University, Washington, DC 20052, United States; 2 Maryland NanoCenter, University of Maryland, College Park, Maryland 20742, United States; 3 Center for Nanoscale Science and Technology, National Institute of Standards and Technology (NIST), Gaithersburg, Maryland 20899, United States

**Keywords:** amplitude-modulation, bimodal, frequency-modulation, multi-frequency atomic force microscopy, multimodal, open loop, trimodal

## Abstract

We present an exploratory study of multimodal tapping-mode atomic force microscopy driving more than three cantilever eigenmodes. We present tetramodal (4-eigenmode) imaging experiments conducted on a thin polytetrafluoroethylene (PTFE) film and computational simulations of pentamodal (5-eigenmode) cantilever dynamics and spectroscopy, focusing on the case of large amplitude ratios between the fundamental eigenmode and the higher eigenmodes. We discuss the dynamic complexities of the tip response in time and frequency space, as well as the average amplitude and phase response. We also illustrate typical images and spectroscopy curves and provide a very brief description of the observed contrast. Overall, our findings are promising in that they help to open the door to increasing sophistication and greater versatility in multi-frequency AFM through the incorporation of a larger number of driven eigenmodes, and in highlighting specific future research opportunities.

## Introduction

**Figure 1:**
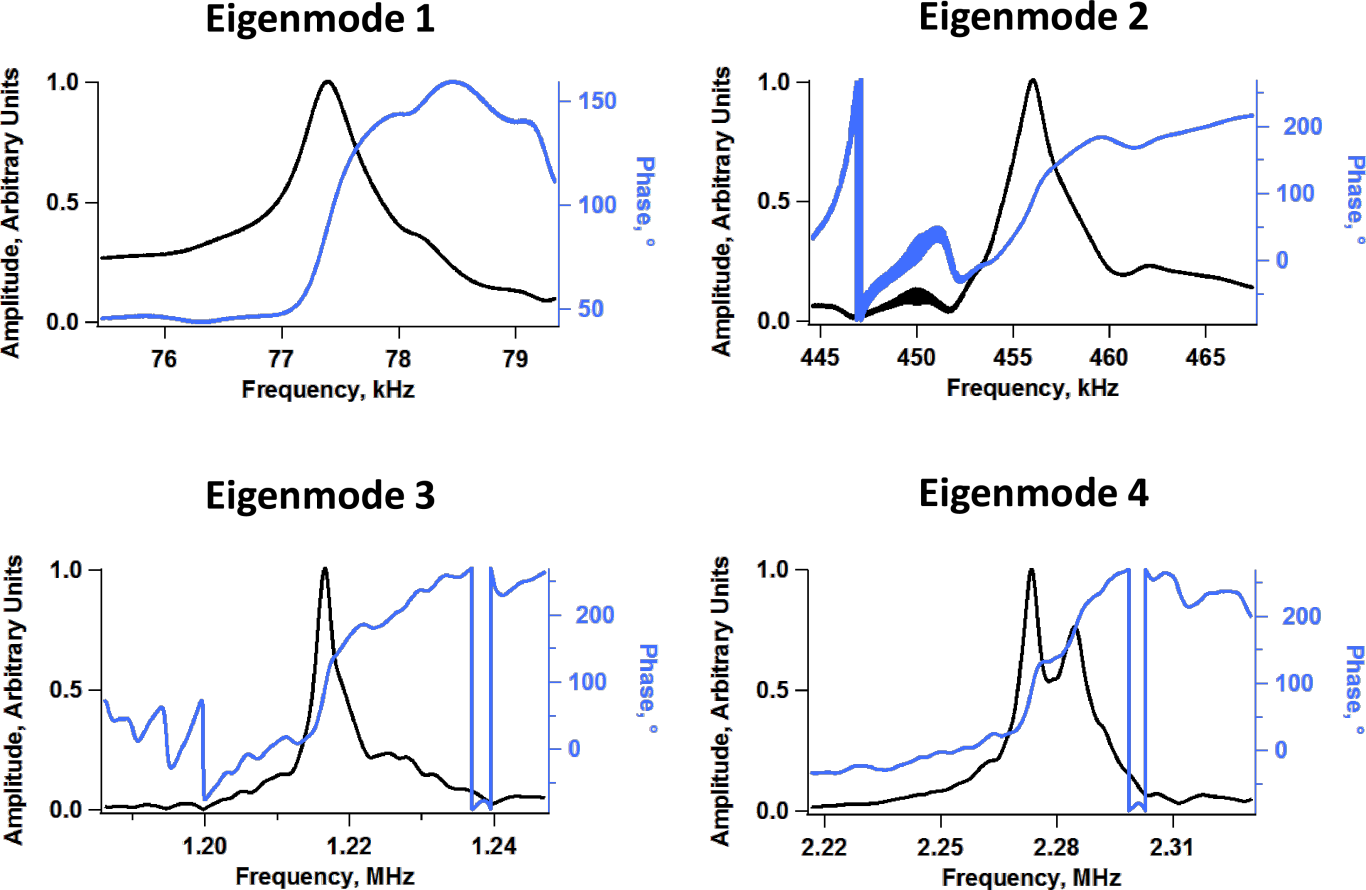
Example of measured frequency response of the first four eigenmodes of one of the rectangular cantilevers used in our experiments, which have nominal fundamental resonance frequency and force constant of 70 kHz and 2 N/m, respectively. As the mode order increases the shape of the peak increasingly deviates from the ideal response of a damped harmonic oscillator.

Multi-frequency atomic force microscopy (AFM) refers to a family of techniques in which the microcantilever probe is driven simultaneously or sequentially at more than one frequency [1]. Often these frequencies correspond to different cantilever eigenmodes [2–12], but there are also methods involving single-eigenmode multi-frequency excitation [13–15] and spectral inversion methods in which the cantilever is driven at a single frequency but the response is analyzed for a range of frequencies [16–18]. Generally speaking, the dynamics of the tip motion become increasingly complex in the case of *simultaneous* multi-frequency excitation, as has been previously reported for multi-eigenmode methods [19–22], which are of particular interest since their purpose is to carry out multiple characterization functions at the same time. Specifically, bimodal AFM methods were developed to perform simultaneous topographical imaging and compositional mapping [2–3], and trimodal methods were later introduced to add imaging depth modulation capability to the bimodal schemes [9]. Although there is not yet an obvious need for methods involving more than three eigenmodes, and although a number of challenges are expected in terms of cantilever quality and drive systems performance (see Figure 1 for an example of non-ideal amplitude vs frequency responses for different eigenmodes), signal processing instrumentation (higher eigenmodes have higher frequencies and require faster electronics as well as tip tracking systems with higher performance), and dynamic complexity [19–22], it is important to explore the feasibility of imaging with multimodal drives since the rapid growth of multi-frequency methods suggests they will soon be of interest [1] (in this paper we use the term multimodal to designate imaging schemes involving more than three eigenmodes).

In general, multimodal imaging can be accomplished with similar equipment to that used for bimodal and trimodal methods [9], except that one needs to include a larger number of oscillation controllers according to the number of active eigenmodes. While the instrumentation is already available, the key open question is whether this type of operation is stable and meaningful. In this paper we explore tetramodal (4-eigenmode) imaging experimentally by using a thin polytetrafluoroethylene (PTFE) film sample and simulate pentamodal (5-eigenmode) cantilever dynamics and spectroscopy computationally (hardware, detection bandwidth and data acquisition limitations prevent us from using the same number of eigenmodes and range of eigenfrequencies in the experiments as in the simulations). We focus on the case of large amplitude ratios between the fundamental eigenmode (used for topographical imaging) and the higher eigenmodes, as in previously validated bimodal and trimodal methods [2–9]. Although the dynamics of multimodal tapping-mode AFM can be quite complex, we find that imaging can be remarkably stable and that the cantilever eigenmodes, in general, exhibit the predicted behavior [20]. We focus our results and discussion section on five different topics, namely tip response in time and frequency space, amplitude and phase response, eigenmode frequency sweep response, imaging, and optimization of the tip–sample impact. We discuss primarily the dynamics and stability of the method and do not offer an interpretation of the additional contrast channels in terms of material properties, as there still remain important open questions even for the bimodal and trimodal methods [20–23]. Overall, our findings are promising and open the door to increasing sophistication and greater versatility in multi-frequency AFM through the inclusion of a larger number of driven eigenmodes along with the corresponding additional contrast channels.

## Results and Discussion

### Tip response in time- and frequency-space

**Figure 2:**
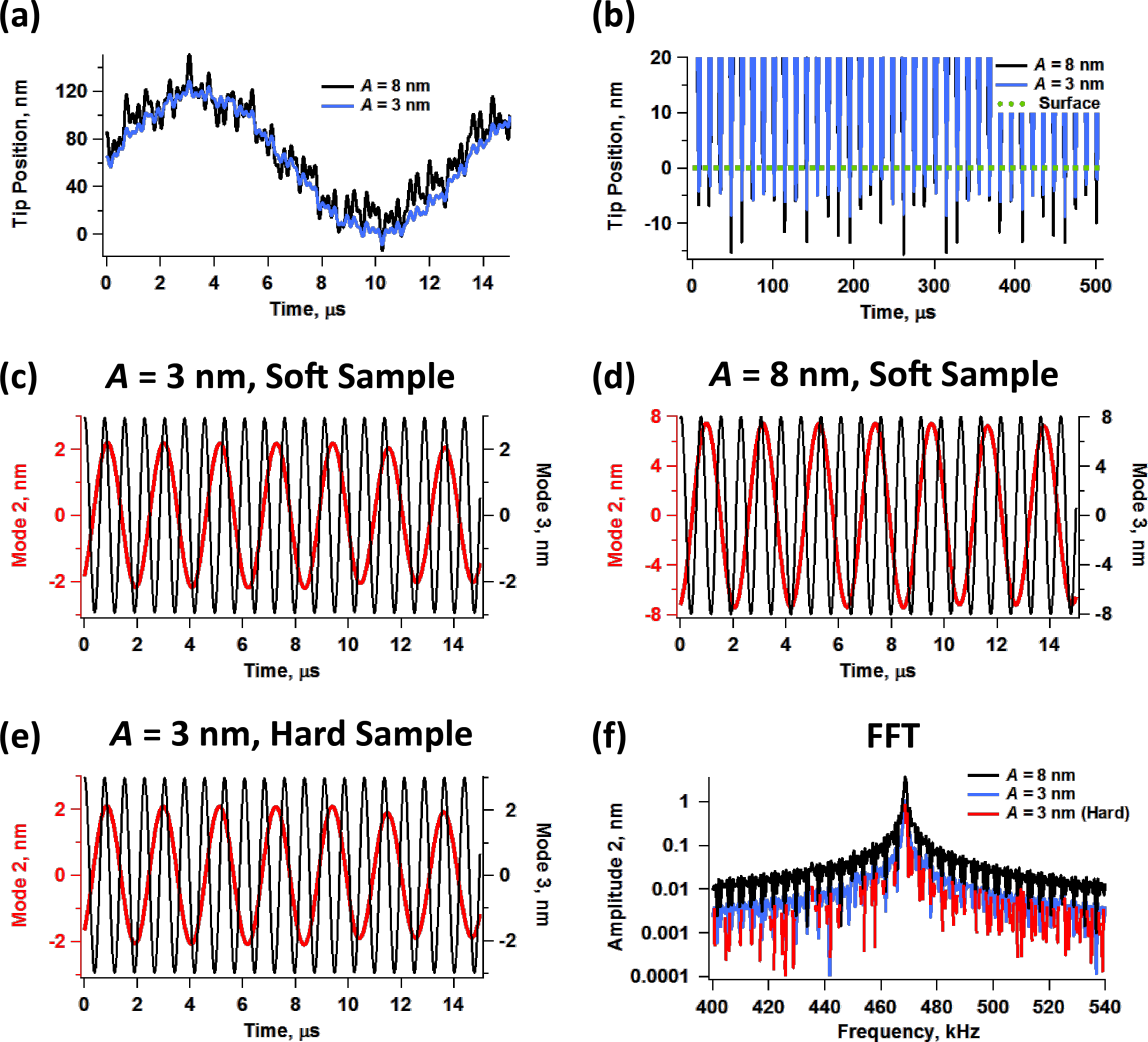
Simulated tip and eigenmode responses for pentamodal tapping-mode AFM: (a) tip trajectories for two different higher eigenmode amplitudes over one fundamental cycle; (b) tip trajectories for two different higher eigenmode amplitudes over multiple fundamental cycles (only the lowest portion of the trajectory is shown, closest to the surface); (c–e) second and third eigenmode trajectories for different free amplitudes and sample parameters; (f) second eigenmode spectra for different free amplitudes. The surface properties were accounted for through a standard linear solid model (see methods section) with *K*_0_ = 7.5 N/m, *K*_inf_ = 7.5 N/m and *C*_d_ = 1 × 10^−5^ N·s/m for the “soft” sample and *K*_0_ = 15 N/m, *K*_inf_ = 15 N/m and *C*_d_ = 3 × 10^−5^ N·s/m for the “hard” sample. The cantilever parameters are ν_1_ = 75 kHz, *k*_1_ = 4 N/m, *Q*_1_ = 150, *Q*_2_ = 3 *Q*_1_, *Q*_3_ = 5 *Q*_1_, *Q*_4_ = 7 *Q*_1_ and *Q*_5_ = 9 *Q*_1_. The free oscillation amplitudes were *A*_1_ = 80 nm, and *A*_2_ = *A*_3_ = *A*_4_ = *A*_5_ = 3 or 8 nm, as indicated on the graphs. The higher mode frequencies and dynamic force constants were scaled by using the eigenfrequency ratios of ideal rectangular beams [1]. The cantilever height was kept fixed at 60 nm above the surface. The responses of modes 2 & 3 are color coded with their respective axes in figures (c) through (e).

The dynamic challenges encountered in multimodal tapping-mode imaging are best appreciated by analyzing the time-dependent trajectory of the tip and individual eigenmodes through simulation of ideal cantilevers. Figure 2a illustrates typical tip trajectories simulated for pentamodal operation when imaging a polymer sample. Here the first eigenmode free amplitude is 80 nm and the higher mode free amplitudes are set to either 3 or 8 nm, as indicated on the graphs, which correspond to typical amplitude ratios used in bimodal and trimodal AFM. As the higher mode amplitudes are increased, the tip trajectory has the appearance of becoming increasingly noisy, which occurs in part because the various eigenfrequencies are generally not integer multiples of one another [1]. Figure 2b shows several successive tip trajectories for the same cases, for typical tapping-mode imaging conditions (only the lowest portion of the oscillation is shown, near the sample), illustrating how the tip can penetrate into the surface to different depths every successive impact, which is not surprising given the irregular tip trajectory. Furthermore, the graph shows that impacts become less regular as the higher mode amplitude increases, which is also as expected. Such irregular impacts constantly generate new transients that in turn lead to non-steady-state tip oscillations. These unsettled oscillations are problematic in the development of generalized theories that relate the measurement observables (amplitudes, phases, frequency shifts, etc.) to material properties because the transients depend on the particular sample, probe and parameters used, as well as on noise levels and non-linear intermodal interactions. However, Figures 2c through 2e show that the oscillation of the individual eigenmodes remains remarkably undisturbed. In fact, the perturbation in the third eigenmode (black traces) is not evident to the naked eye. There is a reduction in the amplitude of the second eigenmode (red traces), which is more significant for smaller free oscillation amplitudes, but it is not excessive in any of the cases considered. Some minor irregularity is observed from one oscillation of the second eigenmode to the next one for stiffer samples (Figure 2e), as was the case in previous studies [20], but the response is still well behaved. This is further confirmed by the oscillation spectrum of this eigenmode, shown in Figure 2f, which exhibits a distinct resonance in all cases. Figures 2c through 2e show only the second and third eigenmode responses, since these are the higher modes with the lowest dynamic force constant [1], which makes them more perturbable than the much stiffer and hardly affected fourth and fifth modes.

### Amplitude and phase response

**Figure 3:**
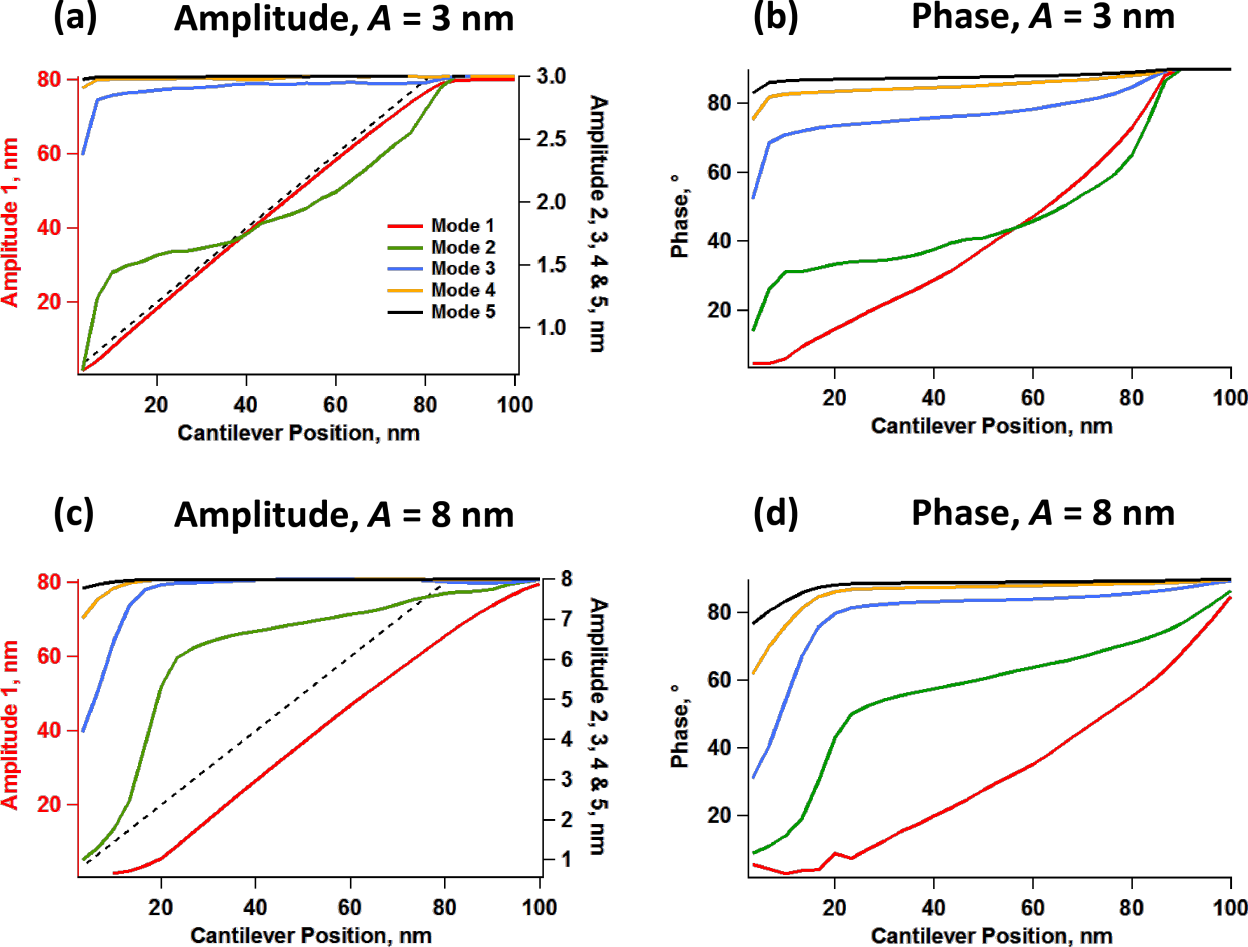
Simulations of amplitude and phase response for two different free amplitudes of the higher eigenmodes. All higher eigenmodes were driven at the same free amplitude in both cases, with the magnitude indicated at the top of the graphs (*A* = 3 nm or 8 nm), while the fundamental free amplitude was set to 80 nm. The simulation parameters are the same as for Figure 2, except that the cantilever position was varied from 100 nm to zero. The dashed lines in graphs (a) and (c) have slope unity and contain the origin, and are provided for guidance.

**Figure 4:**
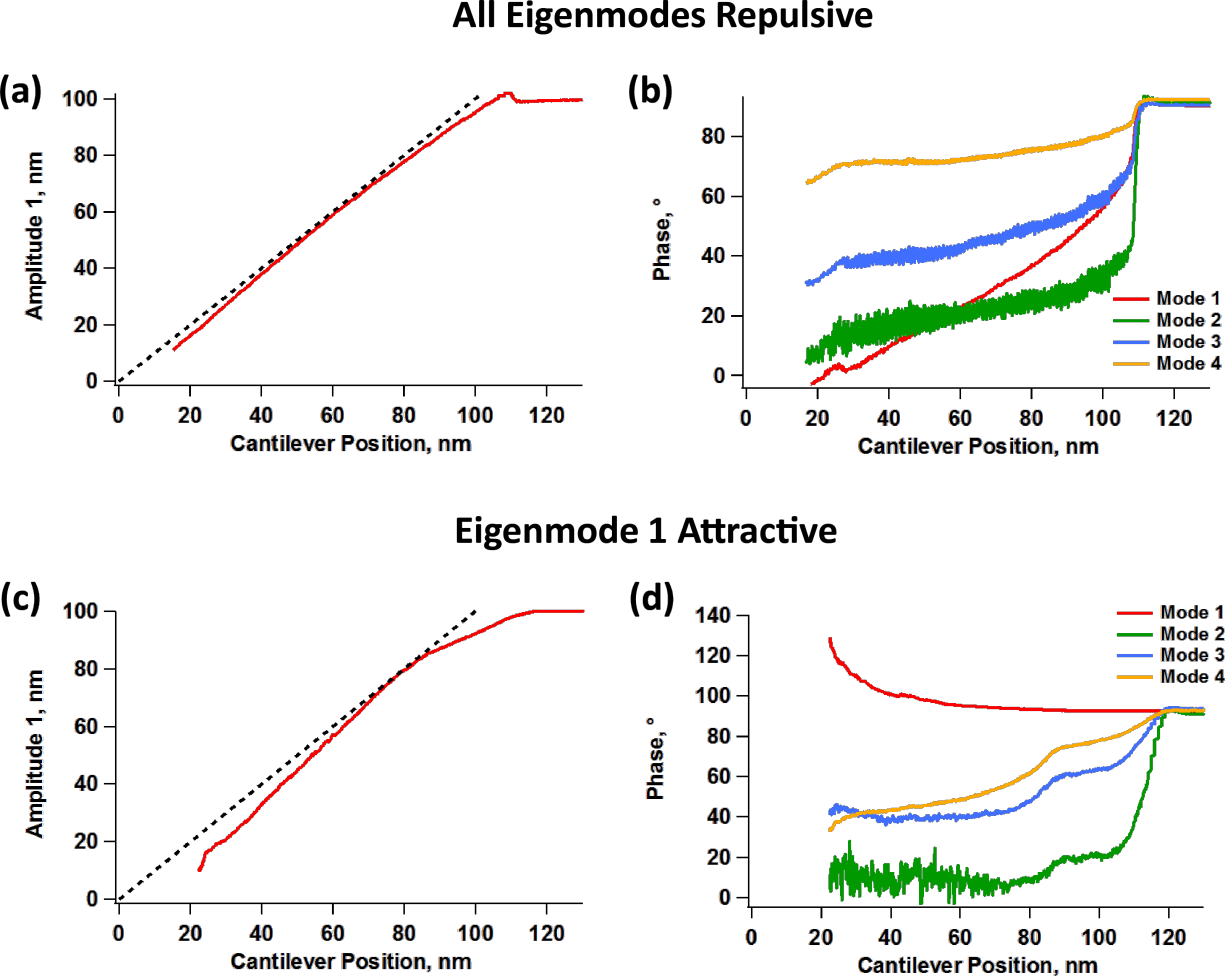
Experimental fundamental amplitude (a, c) and phase responses (b, d) vs cantilever position for tetramodal imaging by using a cantilever similar to the one whose response is illustrated in Figure 1. (a) and (b) correspond to an experiment in which all eigenmodes oscillated in the repulsive imaging regime, similar to the simulations of Figure 3, while (c) and (d) correspond to an experiment in which the fundamental eigenmode oscillated in the attractive imaging regime (the phase of this mode is greater than 90°) while all other eigenmodes oscillated in the repulsive imaging regime. The free oscillation amplitudes for the four eigenmodes were set to approximately 100, 2.9, 1.8, and 1.3 nm, respectively. The dashed lines in graphs (a) and (c) have slope unity and contain the origin, and are provided for guidance.

Simulations of the amplitude and phase behavior for the cases illustrated in Figure 2 show that these key observables also follow the expected trends, as seen in Figure 3 [24]. Figure 3a and Figure 3b provide, respectively, the amplitude and phase response vs cantilever position for pentamodal operation by using higher eigenmode amplitudes of 3 nm, and Figure 3c and Figure 3d provide the corresponding results for higher eigenmode amplitudes of 8 nm (the fundamental free amplitude was set to 100 nm in both cases). The oscillation amplitude of each eigenmode decreases with increasing tip–sample interaction (shorter distance between the cantilever and the sample) and the corresponding phase decreases and deviates increasingly from 90°, indicating in this example a predominantly repulsive interaction [24]. The results also agree with previously known trends [9,19–20] in that the phase and amplitude responses become less and less sensitive as the eigenmode order increases (that is, the magnitude of the phase and amplitude shifts of the higher eigenmodes is in general smaller than for the lower eigenmodes, for the same free oscillation amplitude), and the sensitivity, defined as the rate of change in these observables with respect to a change in cantilever position, also decreases as the higher mode free oscillation amplitude is increased (as discussed in detail in [20] for the trimodal case). This observation is important in terms of signal-to-noise ratio, since the amplitude, phase or frequency shifts, depending on the mode of operation used, could fall below the noise floor for very high (less sensitive) eigenmodes due to their small magnitudes. It is also important to note that although the response curves for different modes exhibit some similarity with one another, they do not necessarily contain the same spectroscopic information, as the shape and curvature can vary significantly from one eigenmode to another, especially for the lowest ones. Different eigenmodes may give different trends in their response variables either due to being able to oscillate in different regimes (attractive or repulsive) with respect to one another or due to nonlinear interactions between them [20–22] (see also Figure 4c and Figure 4d below, which offer an experimental example in which not all eigenmodes oscillate in the same regime). As a result, the spectroscopy theory previously developed for bimodal AFM [25–26] is not necessarily applicable to multimodal AFM. The results of Figure 3 also highlight subtleties in the amplitude curve that could be important depending on the sample and the type of information sought. Specifically, the results show that the amplitude curve for the first eigenmode, which is the basis of the amplitude modulation method [24], is not a straight line as it is in single-mode operation (for guidance, the dashed line in Figure 3a and Figure 3c is a straight line of slope unity, containing the origin). Furthermore, the curvature differs for different higher mode amplitudes (compare the red traces in Figures 3a and 3c) and the curve shifts to the right for larger amplitudes of the higher modes, since the range of oscillation of the tip is the sum of the oscillation ranges of all active eigenmodes.

Figure 4 shows experimental curves analogous to those of Figure 3, but for tetramodal imaging. Since the instrument used has a limited number of acquisition channels, we focused our study on the fundamental amplitude and the four phase responses. Furthermore, since higher eigenmodes have increasingly higher optical detection sensitivity [1], it is possible in an experiment to use smaller physical amplitudes (in units of length) for the highest mode oscillations while still being able to obtain relatively large amplitude readings at the photodetector (in units of voltage), which is also advantageous in terms of making these modes more sensitive [20]. In our experiments we set the free fundamental amplitude to approximately 100 nm and the next three modes to free amplitudes of approximately 2.9, 1.8, and 1.3 nm, respectively. With these settings the amplitude reading at the photodetector for all higher modes was approximately 10% of the reading for the fundamental eigenmode despite the comparatively small physical amplitude of the higher modes. Figure 4a and Figure 4b correspond to an experiment in which all eigenmodes remained in the repulsive regime (as in Figure 3) and Figure 4c and Figure 4d show similar data for an experiment in which the first eigenmode remained in the attractive regime while all others remained in the repulsive regime. Although the simulations assume ideal eigenmode behavior, which is not the case for an experiment (see Figure 1), they do predict important features that were also observed in the experiments. Firstly, non-linear amplitude curves can occur both when the topographical acquisition mode is in the repulsive and in the attractive regime, which as discussed above, has implications for the accuracy of topographical measurement. Second, there is a decrease in the magnitude of the contrast signal (phase shift) as the higher mode order increases, although we did not observe cases in which the highest eigenmode contrast signals fell below the noise floor. Notice also that the second and third phases are noisier than the other two, which is in agreement with the greater propensity of the lower eigenmodes to be perturbed by external forces. Finally, the experimental results illustrate that the curvature of the amplitude or phase response is not necessarily preserved for different eigenmodes (this is especially true when not all eigenmodes operate in the same imaging regime, as illustrated in Figure 4d).

### Engaged frequency response

**Figure 5:**
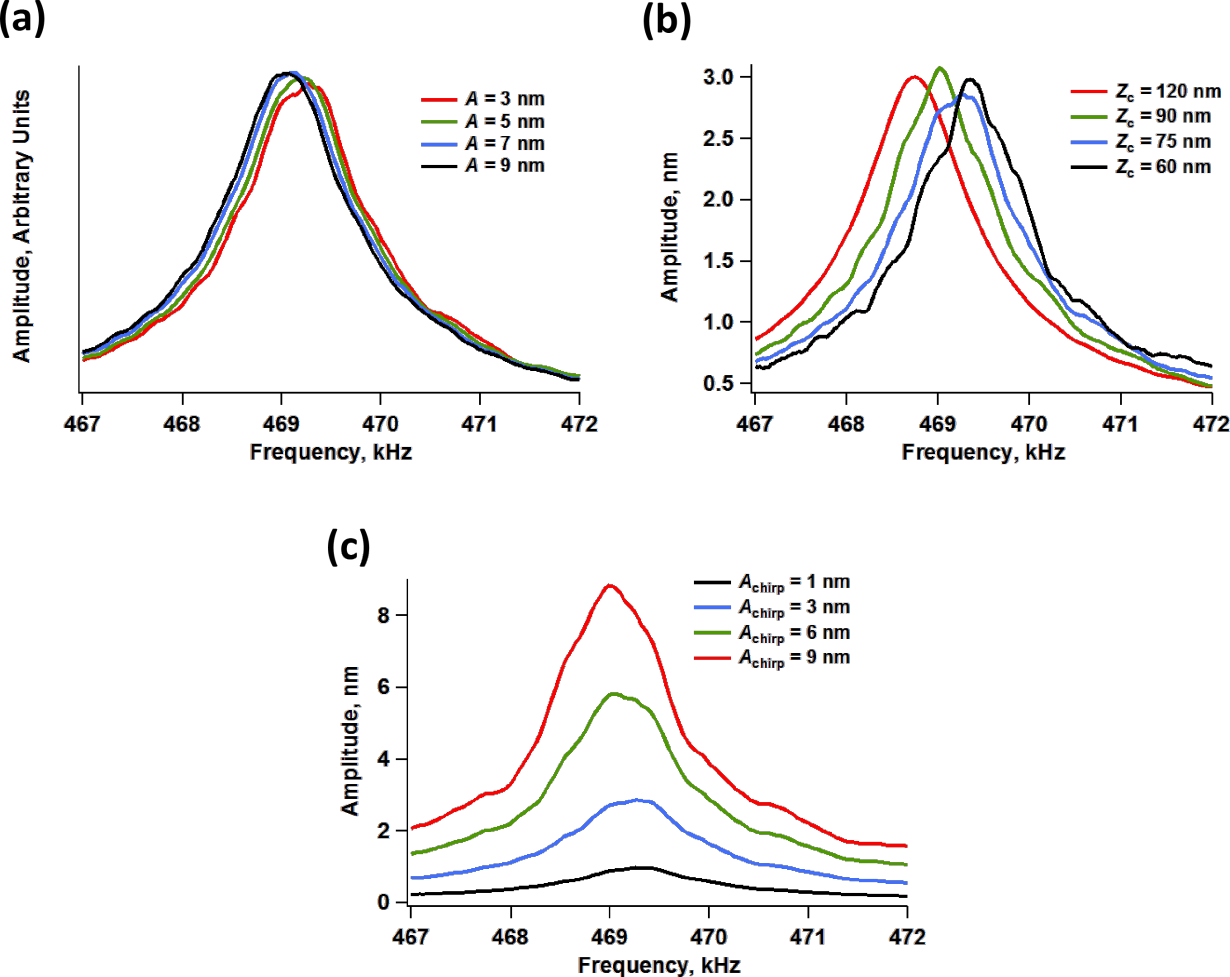
Simulated amplitude vs frequency response of the second eigenmode in pentamodal operation, calculated by sweeping the frequency by using chirp drive functions while the cantilever and sample remained engaged [28]. The simulation parameters are similar to those provided for Figure 2, except that the first eigenmode free amplitude was set to 100 nm. (a) Effect of higher mode free amplitude (*A*_2_ = *A*_3_ = *A*_4_ = *A*_5_ = 3, 5, 7, or 9 nm – Note: the various traces shown are normalized by the free amplitude in each case); (b) effect of cantilever position, *Z*_c_ (*A*_2_ = *A*_3_ = *A*_4_ = *A*_5_ = 3 nm); (c) effect of second eigenmode peak chirp response amplitude (*A*_chirp_ = 1, 3, 6, or 9 nm) for constant amplitude of the highest eigenmodes (*A*_3_ = *A*_4_ = *A*_5_ = 3 nm).

A key consideration regarding the acquisition of meaningful results with multimodal AFM imaging is the quality of the amplitude vs frequency curve of the higher eigenmodes while the cantilever and sample are engaged [20] (this is similar to the usual tuning curve, but with the cantilever and sample engaged). Specifically the degree to which these curves resemble the response of a damped harmonic oscillator, determines the degree to which previous interpretations of the observables and calculated quantities (e.g., phase and amplitude contrast, calculated dissipated power, calculated virial, etc. [25–26]) are applicable. This consideration is also important in cases in which higher modes are excited by using constant drive frequency and amplitude without any feedback (i.e., in ‘open loop’ [2–3]). In such cases, as long as the oscillation is not chaotic, the user will generally be able to obtain an image, but imaging stability does not guarantee that the results are physically meaningful, since it does not guarantee that the contrast eigenmodes conform to the assumed ideal response. In contrast, if frequency modulation methods are used to drive the higher modes [5,7,27], it is necessary that the frequency response be well behaved both to ensure the stability of the controls scheme and to guarantee physically meaningful results. Our simulations show that the highest (least perturbable) eigenmodes retain their ideal response even in multimodal operation. However, it is possible that the response of the lowest eigenmode, excluding the fundamental eigenmode, will be perturbed enough to compromise the stability of a frequency modulation drive. Figure 5 illustrates the amplitude response of the second eigenmode within pentamodal operation when using conditions that are close to those used to construct Figure 2 and Figure 3, for different amplitudes of the higher eigenmodes (Figure 5a), for different cantilever positions above the sample (Figure 5b – the trace for *Z_c_* = 120 nm is the free response), and for different amplitudes of the second eigenmode, while eigenmodes 3 to 5 were driven with a free oscillation amplitude of 3 nm. In the first case (Figure 5a) we observe that the effective resonance frequency of the second eigenmode (location of the peak in each curve) shifts to the left as the amplitude is increased, which is as expected since the influence of the tip–sample forces on the dynamics diminishes for larger amplitudes [20] (here the repulsive tip–sample forces shift the instantaneous resonance frequency of this eigenmode to the right, but this effect diminishes for larger amplitudes, which decrease eigenmode sensitivity [9]). However, the level of perturbation does not change significantly for the range of conditions explored (it only improves slightly for larger amplitudes). In the second case (Figure 5b), we see that the level of perturbation increases, accompanied by a greater frequency shift (due to a greater influence of the repulsive forces in the range of conditions considered), as the cantilever is lowered towards the sample. Additionally, the frequency response curve gradually deviates from the ideal curve, suggesting that the stability of frequency modulation operations may be compromised unless slower scanning speeds are used, which permit greater averaging in the signals. Finally, the results of Figure 5c show that the eigenmode frequency shift increases as its free amplitude is decreased while keeping the other higher amplitudes constant, in agreement with previous results [20] and with Figure 5a, although the shape of the response curve remains distorted for most of the range of amplitudes considered. Despite the nonidealities, the distortions are not extremely drastic for the examples considered, suggesting that frequency modulation drives could still be stable under these conditions as previously shown for trimodal imaging [20]. We recall also that the higher mode responses become more and more regular as the mode order increases (since they are perturbed to a lesser extent by the sample), so the user can in principle select a higher and higher mode whose behavior is close enough to that of an ideal damped harmonic oscillator, in order to enable stable controls. In practice, however, this may not always be feasible or useful in light of the results of Figure 1 which illustrate the typical decline in the quality of the higher eigenmode responses due to tip shaker and cantilever nonidealities.

### Imaging

**Figure 6:**
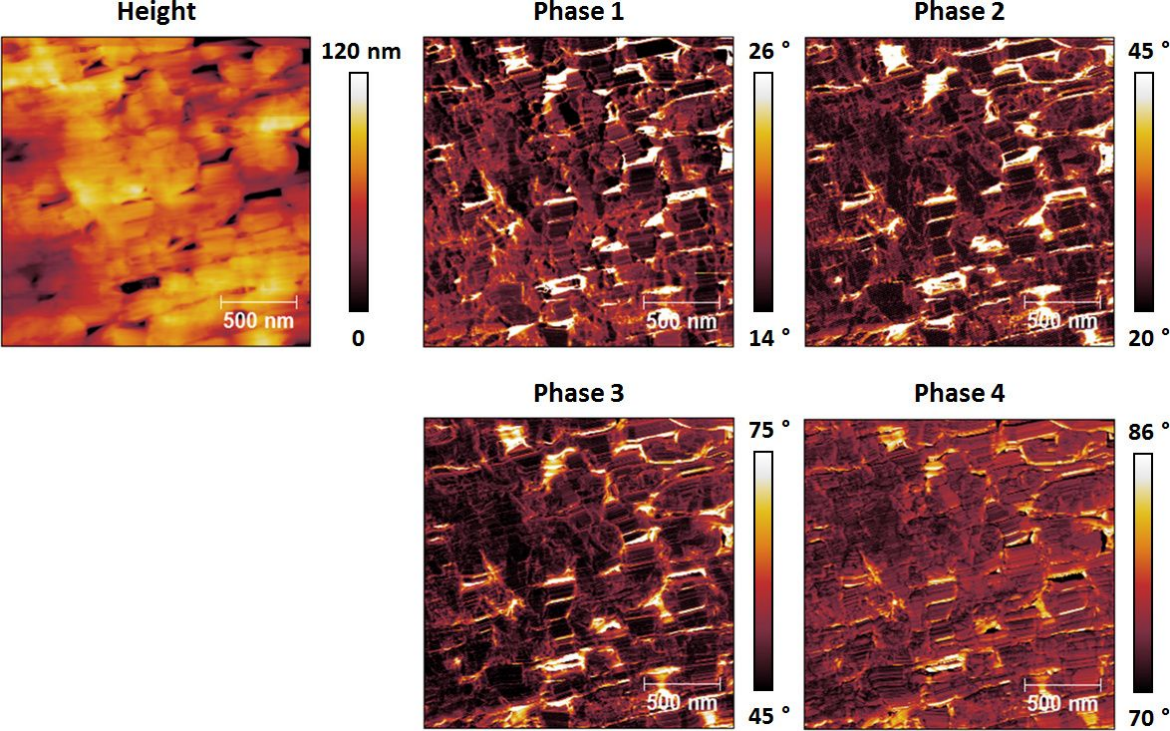
Tetramodal imaging of a thin PTFE film sample by using a cantilever similar to the one whose response is shown in Figure 1. The range of each phase image was chosen such that the contrast is easily discernible. A small percentage of pixels in each image have values that are outside the chosen range. For comparison, Figure 7 shows all phase images of this figure plotted using the same scale. In this experiment all eigenmodes oscillated in the repulsive imaging regime (all phase values are below 90°).

**Figure 7:**
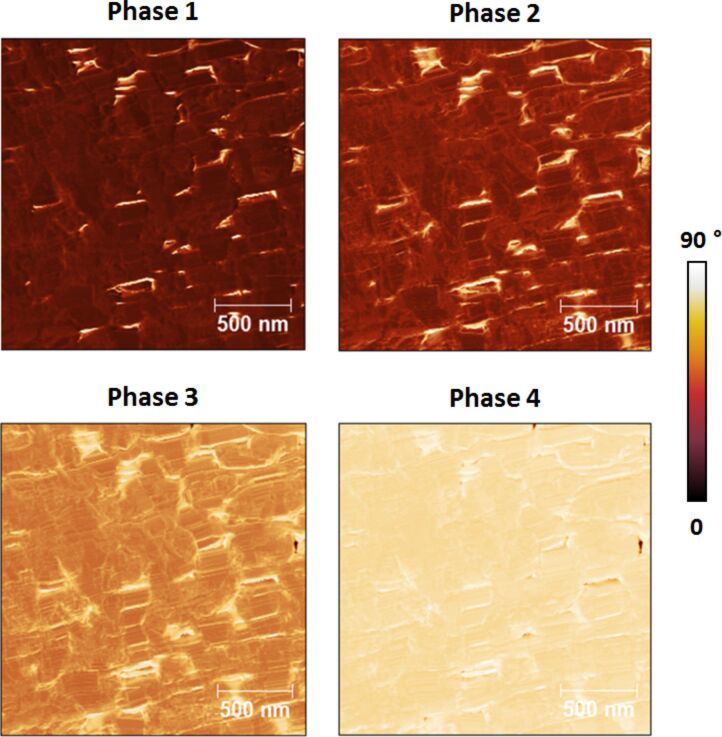
Phase images of Figure 6 plotted using the same scale. As discussed in the text, the phase shifts generally decrease with increasing eigenmode order.

**Figure 8:**
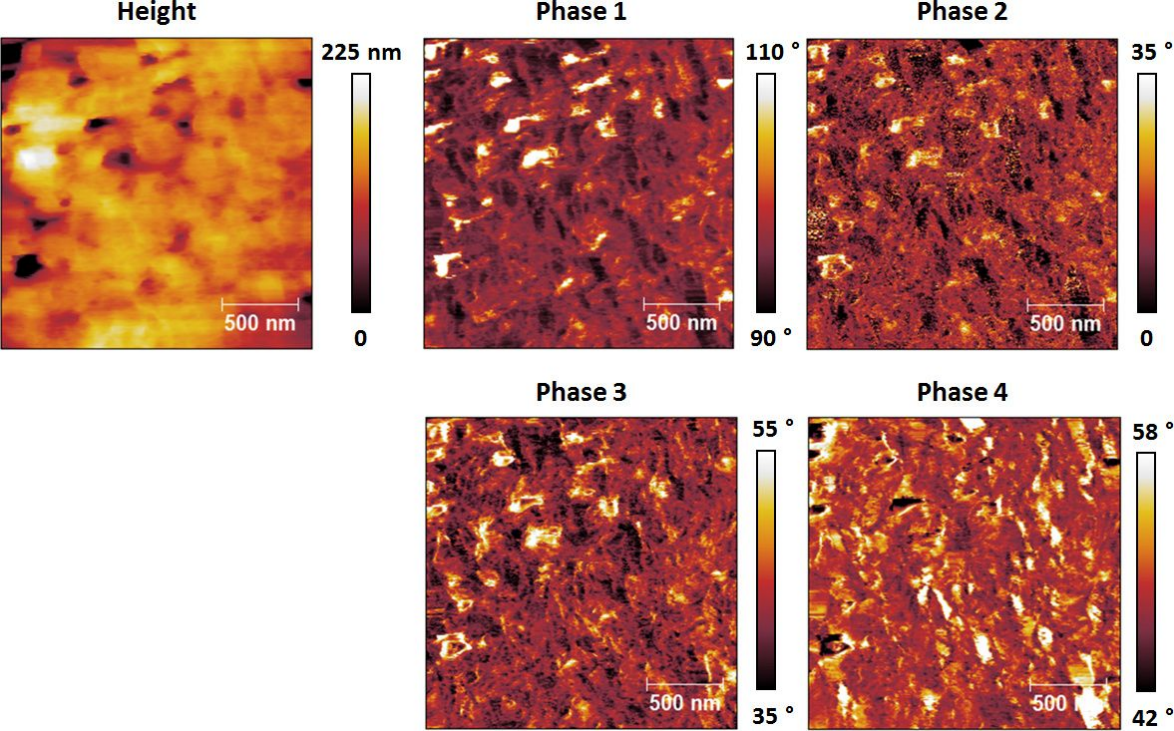
Imaging results analogous to those of Figure 6, but with the first eigenmode oscillating in the attractive regime (the phase values of this mode are greater than 90°).

By using the same settings as for Figure 4, imaging of the PTFE film was carried out by using typical scan rates for tapping mode AFM with an amplitude setpoint ratio of 50 to 60%. Similar to the spectroscopy results of Figure 4, it was possible to image the surface with the fundamental eigenmode operating in the repulsive imaging regime (Figure 6 and Figure 7) as well as in the attractive imaging regime (Figure 8), although there was little control on the selection of the regime. In general, higher free amplitudes, lower amplitude setpoints, and drive frequencies lower than the natural frequency favor the repulsive regime, but the result is also strongly determined by the cleanness and sharpness of the tip. Cleaner and sharper tips undergo smaller tip–sample attraction due to their smaller effective radius of curvature. Therefore, they are more amenable to imaging in the repulsive regime, which in general gives sharper topographical contrast since it is governed by contact as opposed to long-range forces which are more likely to cause tip broadening artifacts [29].

For characterization in the repulsive regime (see Figure 6) the phase images generally resembled one another for all eigenmodes, although differences and even contrast inversion between eigenmodes emerged on certain regions of the sample as the imaging conditions were changed. Additionally, the varying range of phase values observed for each eigenmode (phase range decreasing with increasing mode order) confirms the decreasing sensitivity of higher eigenmodes, even though higher modes were driven with smaller amplitudes (see parameters in the caption of Figure 4). This is better illustrated in Figure 7, where all phase images from Figure 6 are plotted using the same scale. Clearly the highest modes exhibit the smallest phase shift from 90°. In contrast, the phase images acquired driving the fundamental eigenmode in the attractive regime almost always exhibited partial contrast inversion with respect to one another. For example, some bright spots in the first phase image of Figure 8 look dark in the fourth phase image and vice versa. The observed contrast inversion may be related to the mechanism previously identified for bimodal imaging, which was related to the energy content in each eigenmode [20–22], or may be the result of nonlinear interactions between the eigenmodes, given the complexity of the multimodal tip–sample impact.

### Optimization of the tip–sample impact

**Figure 9:**
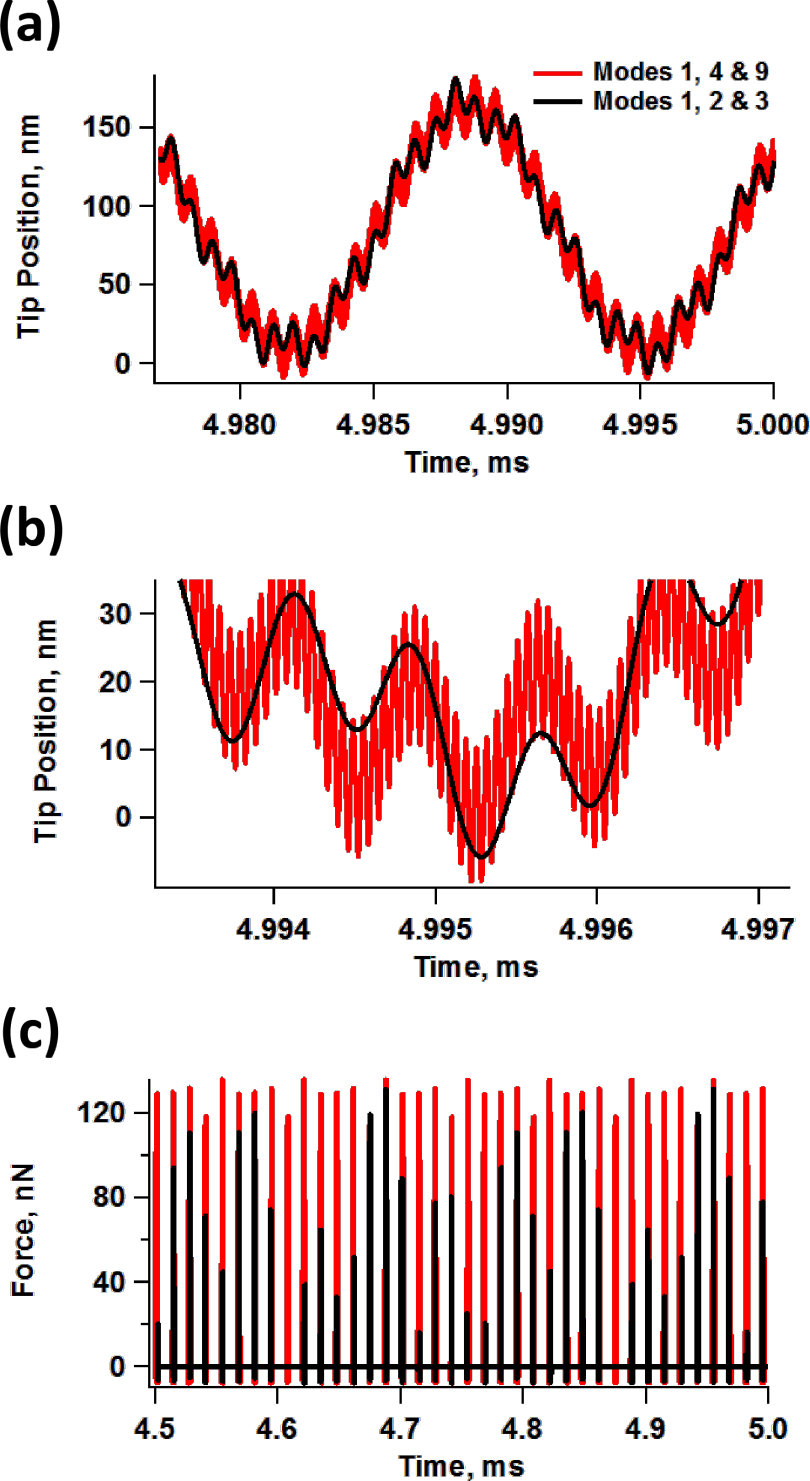
(a) Comparison of tip trajectories for trimodal oscillations using the first three eigenmodes (*A*_1_ = 100 nm, *A*_2_ = *A*_3_ = 3 nm) and eigenmodes 1, 4 and 9 (*A*_1_ = 100 nm; *A*_4_ = *A*_9_ = 3 nm) with *A*_setpoint_ ≈ 80%; (b) close-up of the lowest portion of the tip trajectory for the above cases; (c) illustration of force trajectories for the above cases (notice how the peak forces in successive impacts become more similar to one another as eigenmode spacing increases). The sample and cantilever parameters are the same as for Figure 2.

Despite the stability with which imaging can be carried out and the apparent robustness of our results, the non-uniformity of successive tip–sample impacts, the nonlinear coupling of the various eigenmodes, as well as time-dependent tip–sample behaviors such as viscoelasticity suggest that unless single-cycle techniques [16,18,30] can be implemented accurately for multimodal imaging, it may not be possible to carry out fully quantitative measurements of the surface properties. As already discussed, the tip trajectories for a tetramodal or pentamodal operation are even more complex than the already complex bimodal [19] and trimodal [20] trajectories. Furthermore, the variation of the impact shape from one fundamental oscillation to the next one results in non-steady-state dynamics which may not only require lower scanning speeds in order to properly characterize each location on the surface, but which may also preclude the application of spectroscopy theories based on ideal responses and the recording of observables averaged over multiple cantilever cycles [25–26]. However, our simulations also show that the regularity of the tip–sample impact can be significantly improved by choosing non-consecutive eigenmodes with relatively high frequency ratios among them, as illustrated in Figure 9 for the trimodal case (the regularity of the impact depends on the frequency spacing between each pair of active eigenmodes, so similar arguments can be made for other multimodal cases). Figure 9a compares the trajectories of a trimodal oscillation using eigenmodes 1, 2 and 3 vs using eigenmodes 1, 4 and 9. It is clear that the successive impacts shown differ significantly in the former case, but not in the latter (see also Figure 9b, which shows a close-up view of the lowest portion of the tip trajectories of Figure 9a, illustrating a more symmetric and regular impact when the eigenmode spacing is greater). Figure 9c shows the force trajectory for a large number of impacts, confirming that successive tip–sample interactions become more similar with greater eigenmode spacing. Notice how the peak forces over a large number of fundamental oscillations in Figure 9c are similar in both cases, but the dynamics are less steady for the case when the first three eigenmodes are used. Note also that in order to improve the regularity of the multimodal tip–sample impact, it is necessary to increase the frequency ratio for each pair of adjacent eigenmodes. For the case discussed here, for example, using eigenmodes 1, 8 and 9 would not be as effective as using eigenmodes 1, 4 and 9. Although the spacing between the first two active eigenmodes would be large in the former case, the frequency ratio of the highest two eigenmodes (8 and 9) would only be about 1.3. The results of Figure 9 suggest that it could in some cases be advantageous to maximize the frequency ratios when selecting the active eigenmodes, although this may not always be possible due to bandwith limitations in the electronics and cantilever and shaker non-idealities (see Figure 1). As stated above, the use of very high eigenmodes can also result in low signal-to-noise ratios due to the decreasing sensitivity in the spectroscopic observables (e.g., small phase shifts, etc.) with increasing mode order, despite the higher optical sensitivity in tracking the tip response [1].

## Conclusion

We have explored experimentally and computationally the dynamics and stability of multimodal tapping-mode atomic force microscopy when using more than three active cantilever eigenmodes. We have illustrated the increase in complexity with a larger number of eigenmodes, as well as its indirect effect on the topographical measurement and the response of the spectroscopic observables. We have also shown that stable imaging is possible, although contrast differences emerging from the nonlinear interaction of the eigenmodes are also expected. Overall our findings are positive and encourage further development of multimodal techniques, as well as fundamental research on the probe dynamics and on the measurement process itself. We take the opportunity to remind the reader that our results are only applicable to measurements performed in air environments, corresponding to fundamental quality factors of a few hundreds, and that the work reported here represents by no means an exhaustive study. High-damping environments may offer even greater complexities [31] and our amplitude-modulation/open-loop results are not directly applicable to vacuum environments [24,32].

## Methods

### Experimental

The tetramodal experiments were performed using a Cypher AFM (Asylum Research, Santa Barbara, CA), driving all four modes through the internal lock-ins of the instrument (see disclaimer below). Since the number of signals that could be recorded was limited to six, we recorded all the eigenmode phases along with the fundamental amplitude during the experiments. Images were acquired with a resolution of 512 × 512 pixels at a scan rate of 1 Hz in the fast direction. We used a commercial cantilever having a nominal fundamental resonance frequency of 70 kHz and a nominal fundamental force constant of 2 N/m. The sample consisted of PTFE pipe thread seal tape (nominal thickness ca. 0.1 mm) stretched onto the back of single-sided scotch tape, which was adhered sticky side down onto a metal substrate. This type of polymer sample was chosen in order to obtain high contrast in the phase signals.

### Computational

For the numerical simulations five eigenmodes of the AFM cantilever were modeled by using individual equations of motion for each, coupled through the tip–sample interaction forces as in previous studies [8,20]. Driven eigenmodes were excited through a sinusoidal tip force of constant amplitude, and frequency equal to the natural frequency. Chirp excitation functions [8,28] were used to construct the engaged amplitude vs frequency curves of Figure 5. The equations of motion were integrated numerically and the amplitude and phase of each eigenmode were calculated using the customary in-phase (*I_i_*) and quadrature (*Q_i_*) terms:

[1]
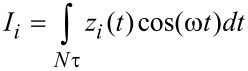


[2]
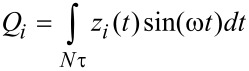


where *z_i_*(*t*) is the spatial response of the *i*th eigenmode in the time domain, *N* is the number of periods over which the phase and amplitude were averaged, ω is the excitation frequency, and τ is the nominal period of one oscillation. The amplitude (*A_i_*) and phase (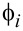) were calculated, respectively, as:

[3]
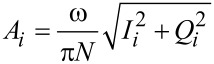


[4]
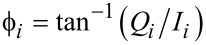


**Figure 10:**
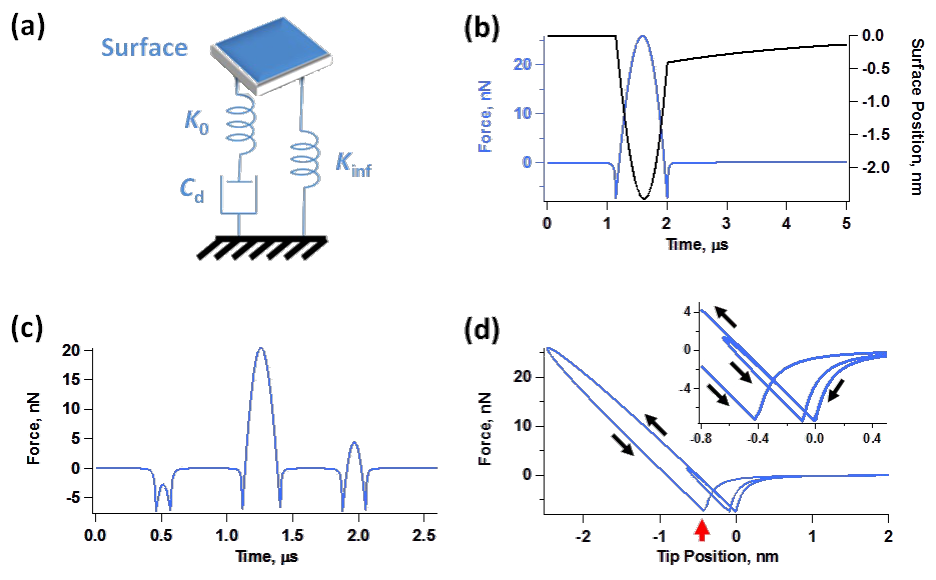
(a) Standard linear solid (SLS) model [9]; (b) illustration of the force trajectory for a single tip–sample impact along with the relaxation trajectory of the surface (notice how the surface remains temporarily indented and gradually recovers after the impact); (c) illustration of a triple impact within a single cycle of the fundamental oscillation for a multimodal imaging case (see also the discussion on multiple impacts in reference [20]); (d) force curve (force vs tip position) for a double-impact, illustrating also the temporary depression of the surface due to relaxation of the SLS model (the red arrow illustrates the depressed position of the surface where tip–sample contact is lost after the first impact).

The repulsive tip–sample forces were accounted for through a standard linear solid (SLS) model [9] which exhibits both stress relaxation and creep (see Figure 10 and notice the variety of force and surface trajectories for the single and multiple impacts observed in multimodal tapping-mode imaging [20]). Long-range attractive interactions were included via the Hamaker equation [24] for a tip radius of curvature of 10 nm and a Hamaker constant of 2 × 10^−9^ J.

### Disclaimer

Certain commercial equipment, instruments or materials are identified in this document. Such identification does not imply recommendation or endorsement by the National Institute of Standards and Technology, nor does it imply that the products identified are necessarily the best available for our purposes.
